# Cytokines impact natural killer cell phenotype and functionality against glioblastoma *in vitro*

**DOI:** 10.3389/fimmu.2023.1227064

**Published:** 2023-09-28

**Authors:** Minna Sivonen, Katja A. Sirviö, Sara Wojciechowski, Anssi Kailaanmäki, Satu Kaipainen, Aubrey Bailey, Martin Villalba, Tuija Kekarainen

**Affiliations:** ^1^ Kuopio Center for Gene and Cell Therapy, Kuopio, Finland; ^2^ A.I. Virtanen Institute, Biotechnology and Molecular Medicine Unit, University of Eastern Finland, Kuopio, Finland; ^3^ IRMB, University of Montpellier, INSERM, CNRS, CHU Montpellier, Montpellier, France; ^4^ A.I. Virtanen Institute for Molecular Sciences, Faculty of Health Sciences, University of Eastern Finland, Kuopio, Finland

**Keywords:** immunotherapy, natural killer cells, glioblastoma, cytokines, memory-like natural killer cells

## Abstract

**Objective:**

Natural killer (NK) cells are a part of the innate immune system and first-line defense against cancer. Since they possess natural mechanisms to recognize and kill tumor cells, NK cells are considered as a potential option for an off-the-shelf allogeneic cell-based immunotherapy. Here, our objective was to identify the optimal cytokine-based, feeder-free, activation and expansion protocol for cytotoxic NK cells against glioblastoma *in vitro*.

**Methods:**

NK cells were enriched from human peripheral blood and expanded for 16 days with different activation and cytokine combinations. The expansion conditions were evaluated based on NK cell viability, functionality, expansion rate and purity. The cytotoxicity and degranulation of the expanded NK cells were measured *in vitro* from co‑cultures with the glioma cell lines U‑87 MG, U‑87 MG EGFR vIII, LN-229, U-118 and DK-MG. The best expansion protocols were selected from ultimately 39 different conditions: three magnetic cell‑selection steps (Depletion of CD3+ cells, enrichment of CD56+ cells, and depletion of CD3+ cells followed by enrichment of CD56+ cells); four activation protocols (continuous, pre-activation, re-activation, and boost); and four cytokine combinations (IL-2/15, IL‑21/15, IL‑27/18/15 and IL-12/18/15).

**Results:**

The expansion rates varied between 2-50-fold, depending on the donor and the expansion conditions. The best expansion rate and purity were gained with sequential selection (Depletion of CD3+ cells and enrichment of CD56+ cells) from the starting material and pre-activation with IL‑12/18/15 cytokines, which are known to produce cytokine-induced memory-like NK cells. The cytotoxicity of these memory-like NK cells was enhanced with re-activation, diminishing the donor variation. The most cytotoxic NK cells were produced when cells were boosted at the end of the expansion with IL-12/18/15 or IL-21/15.

**Conclusion:**

According to our findings the *ex vivo* proliferation capacity and functionality of NK cells is affected by multiple factors, such as the donor, composition of starting material, cytokine combination and the activation protocol. The cytokines modified the NK cells' phenotype and functionality, which was evident in their reactivity against the glioma cell lines. To our knowledge, this is the first comprehensive comparative study performed to this extent, and these findings could be used for upscaling clinical NK cell manufacturing.

## Introduction

Glioblastoma (GBM) is a highly aggressive brain tumor originating from the nerve cell-supporting astrocytes. Current treatments for GBM include surgical removal of the tumor, radiation, chemotherapy, and monoclonal antibodies (mAbs) (1). These therapies may slow down the progression and reduce symptoms, but curative treatments for GBM are not available. Adoptive cell therapies using immune effectors, such as CD56+/CD3- lymphocytes known as natural killer (NK) cells, provide promising new approaches to treat hematological and solid malignancies. The presence of NK cells at the tumor site has been shown to correlate with better prognosis, suggesting their important role in cancer prevention and immunosurveillance (2). As an effector lymphocyte of the innate immune system, the NK cell possesses a wide range of tumor recognition and killing mechanisms and are an essential part of the first line defense against malignant cells. On their arrival to the tumor site NK cells get activated by soluble (e.g. IL-2, IL-15, TNF-α, and IFN-γ) and contact-dependent signals from the tumor microenvironment (TME). NK cells respond to stress signals such as NKG2D ligands [MHC class I chain-related proteins (MICA/MICB) and UL16 binding proteins (ULBP-1 to ULBP-6)], to nectins and nectin-like (Necls) molecules with DNAM-1 (3, 4), or to an absence of self-antigens such as downregulation of HLA class I (5–8). NK cells mediate their cytotoxic activity by releasing cytotoxic granules containing perforin and granzymes, or via death receptor-mediated apoptosis by TNF-related apoptosis-inducing ligand (TRAIL) and/or Fas ligand (FasL), which engage TRAIL-R1/R2 or Fas, respectively, on the target cell. NK cells can also induce antibody-dependent cell mediated cytotoxicity (ADCC) via FcɣRIIIa (also known as CD16) receptor against opsonized cells (9). In addition to their innate killing mechanisms NK cells provide the possibility for an allogeneic off-the-shelf clinical application as they do not require strict HLA matching or carry the risk of graft-versus-host disease (GvHD) (10, 11). However, low numbers of NK cells in the peripheral blood [5–20% of the circulating lymphocytes (12)] bring challenges to the manufacturing of NK cell-based therapies. This has led to various approaches to expand NK cells *ex vivo*. The starting material for *ex vivo* expansion is typically peripheral or umbilical cord blood combined with depletion of CD3+ cells, or T cell suppression [anti-CD3 (OKT3)], with or without enrichment of CD56+ cells (13–16). Most expansion protocols use cytokines or co-culturing with autologous accessory or growth-inactivated feeder cells (14, 17–19). Although accessory or feeder cell-based expansion protocols have been reported to yield high NK cell numbers, they complicate the manufacturing process and require extensive quality controls (20, 21). The most commonly used cytokines are IL-2 and/or IL-15, and also cytokines IL-12, IL-15, IL-18, IL-21, and IL-27 have been utilized in NK cell expansions (12, 18). IL-12, IL-18, and IL-21 are known to strengthen NK cell cytotoxicity (22), IL-27 promotes NK cell effector function and enhances IL-15/18 mediated activation (23, 24), and pre-activation with IL-12, IL-18 and IL-15 is known to induce cytokine-induced memory-like (ML) NK cells. These ML-NK cells are long-lived and respond more potently to a variety of triggers, such as cancer cells (25, 26).

Here, we discovered that depletion of CD3+ cells followed by enrichment of CD56+ cells resulted in the highest purity in NK cell expansion. Cytokine combinations (IL-2/15, IL-21/15, IL-12/18/15, IL-27/18/15) and activation timing (pre-activation, re-activation, boost or continuous) had a significant impact on the phenotype, expansion, and functionality of *ex vivo* expanded NK cells against a variety of glioblastoma cell lines *in vitro*. Despite prior studies on the different selection methods, cytokine combinations, and timing of activation, no comparative studies on this topic have been published to date.

## Materials and methods

### *Ex vivo* expansion of NK cells

Human peripheral blood mononuclear cells (PBMCs) were isolated from peripheral blood products by density gradient centrifugation (Histopaque^®^-1077 HybriMax™). Depletion of CD3+ cells was achieved using CD3 MicroBeads and LD columns, and enrichment of CD56+ cells with CD56 MicroBeads and LS columns. MACS^®^ MultiStand and QuadroMACS™ Separator was used for magnetic separation (all from Miltenyi Biotec).

Three selection methods [Depletion of CD3+ cells (CD3-), enrichment of CD56+ cells (CD56+), and their combination (CD3-/CD56+)], four activation protocols (continuous, pre-activation, re-activation, and boost) and four cytokine cocktails [IL-21 (25 ng/ml) + IL-15 (10 ng/ml); IL-15 (10 ng/ml) + IL-18 (50 ng/ml) + IL-12 (10 ng/ml); IL-15 (10 ng/ml) + IL-18 (50 ng/ml) + IL-27 (10 ng/ml); IL-2 (100 IU/ml) + IL-15 (10 ng/ml)] were used for the expansions (**Supplementary Figure 1**). Concentrations were based on the following publications and preliminary studies: IL-2 (14), IL-15 (24, 27), IL-21 (27), IL-12 and IL-18 (25, 28), and IL-27 (24). The following cytokines were used: premium grade recombinant human (rh) IL-2 Improved Sequence (IS), rhIL-12, rhIL-15; research grade rhIL-21, rhIL-27 (all from Miltenyi Biotec), and rhIL-18 (InvivoGen). NK cells were cultured in RPMI-1640 medium (Lonza) supplemented with 5% human AB serum (HABS; Sigma-Aldrich), 2 mM GlutaMAX™ (Gibco), 100 U/ml penicillin and 100 µg/ml streptomycin (P/S, Gibco) (hereinafter referred to as complete medium). Cells were cultured in a humidified atmosphere at 37°C, with 5% CO_2_ for 16 days. Day 16 was selected based on previous studies (14, 29, 30) and preliminary experiments (unpublished data). Expansions were started in T25 flasks, cells were counted and complete medium added, or partly changed, every 2-3 days and the cells were kept approximately at 1x10^6^ cells/ml during expansion. Cell count and viability were determined using NucleoCounter^®^ NC 200™ automated cell counter (ChemoMetec), or Guava MUSE^®^ cell analyzer using Muse Count & Viability Kit (Luminex).

Used activation protocols are illustrated in **Supplementary Figure 1**: Pre-activation (Pre) was done as previously in (25) with 2x10^6^ cells/ml density for 16-18 hours with IL-21/15, IL-27/18/15 or IL-12/18/15, after which the medium was changed to complete medium with 10 ng/mL IL-15, and the cell concentration was adjusted to 1x10^6^/ml. Re-activation (Re) started on day 15 (16-18 hours before harvest), with same cytokines used for pre-activation. Boosted cells (Boost) were expanded in complete medium with 10 ng/ml IL-15 and the boosting cytokines (IL-21/15, IL-27/18/15, or IL-12/18/15) were added 16–18 hours before harvest on day 16. For the continuous group (Cont), the cytokines were added every 2-3 days with the fresh complete medium.

### Target cell lines

DK-MG (ACC277, glioblastoma multiforme) was obtained from German Collection of Microorganisms and Cell Cultures (DSMZ), U-87 MG human EGFR vIII (CL01004-CLTH, epithelial, likely glioblastoma, transduced with epidermal growth factor receptor variant III (EGFR vIII), hereinafter referred to as U87vIII) from Amsbio, U-87 MG (HTB-14, epithelial, likely glioblastoma, hereinafter referred to as U87-wt), LN-229 (CRL-2611, epithelial, glioblastoma) and U-118 MG (HTB-15, mixed, grade IV glioblastoma, hereinafter referred to as U-118) both from ATCC. U87-wt, U87vIII, LN-229, and U-118 were maintained in DMEM (high glucose and pyruvate, Gibco) supplemented with 10% fetal bovine serum (FBS, gamma irradiated, Gibco) and 100 U/ml penicillin and 100 µg/ml streptomycin (P/S). In addition to this, U87vIII medium was supplemented with 200 µg/ml of the selection antibiotic Geneticin (Gibco). DK-MG were maintained with RPMI-1640 supplemented with 10% FBS, P/S, and 2 nM GlutaMax. Cells were passaged when they reached 80-90% confluency.

### Flow cytometric characterization

Cells were harvested and washed with FACS Buffer [phosphate buffered saline (PBS, Gibco) + 0.02% Sodium Azide (NaN_3_, Sigma) + 2 mM ethylenediaminetetraacetic acid (EDTA, Sigma) + 1% bovine serum albumin (BSA, Biomol)]. Before staining, samples were blocked with 10% human FcR Blocking Reagent (Miltenyi Biotec) in FACS buffer. Antibody panels were added to the cells for 15 min at RT then washed with FACS Buffer (centrifugation 300 x g, 6 min). The following anti-human mAbs were used (indicated as target and fluorochrome): CD45-APC Fire750, CD3-BV510, and CD56-BV421 to evaluate the NK cell purity; CD25-PE, CD16-VB515, NKp46-PECy7, and CD69-APC as NK cell phenotype markers; ITGAL-FITC, TRAIL-APC, NKG2D-BV605, and FasL-PE as markers for functionality; MIC-A/B-PE, ULPB-1-PE, ICAM-1-APC, and EGFR-APC for target phenotyping. Used clones are listed in **Supplementary Table 1**. All fluorescent mAbs were obtained from Biolegend, except the CD16 mAb from Miltenyi Biotech and EGFR from Absolute Antibody. 7-amino-actinomycin D (7-AAD) was used in all panels to identify the live and dead cells (BD Biosciences). Cells were run on CytoFLEX S (Beckman Coulter) and analyzed with CytExpert v2 (Beckman Coulter) and FlowJo v10 (BD Life Sciences, TreeStar).

### Cytotoxicity assay

Cytotoxicity was determined from the co-cultures with Cell Counting Kit-8 (CCK-8; CK04-13, Dojindo). Target glioma cells (T) were seeded into flat 96-well plates at a density of 2 x10^4^ cells per well, in cell type-specific medium (see above). Cells were allowed to settle in wells at room temperature (RT) for 15 minutes before 24 hours incubation at 37°C, 5% CO_2_. After 24 hours, the medium was discarded and NK cells (Effectors; E) were added in RPMI-1640 medium supplemented with 5% HABS, 2 mM GlutaMAX and 100 U/ml penicillin 100 µg/ml streptomycin, without cytokines. Three effector to target (E:T) cell ratios (0.5:1, 1:1, and 2:1) were used. E:T ratios were calculated based on the amount total viable effector cells. In some experiments, additional cytokines, 5 ng/ml IL-15 or 20 IU/ml IL-2 were added to the co-cultures. After 24 hours co-culture the target cell viability was determined with CCK-8 [10% CCK-8 in RPMI-1640 without phenol red (Lonza)] following the manufacturer’s instructions, with 1 hour incubation. Absorbance was measured at 450 nm with Cytation5 (BioTek) multi-mode reader. After blank subtraction the relative cytotoxicity was calculated against the control (targets only) wells as follows:


$$Cytotoxicity\;\%=\frac{ODt-ODte}{ODt}x\;100\%\;\;$$

OD=Absorbance;ODt=Target alone;ODte=Target+effectors

### NK degranulation assay

To analyze NK cell degranulation, CD107a expression was measured before (baseline expression) and after co-culturing with target cells by flow cytometry. Staining was done as in flow cytometric characterization. For the co-culturing, 1.5x10^5^ target glioma cells were seeded per well into 24-well plates. After 24 hours, the NK cells were added in 1:1 E:T ratio, and after 3 hours co-culturing the cells were collected and stained with CD45-APC-Fire750, CD107a-PE (Miltenyi Biotec) and 7-AAD. CD107a expression was measured from viable CD45 positive population with CytoFLEX S and analyzed with CytExpert v2 and FlowJo v10.

### Statistical analysis

Statistical analyses were performed using GraphPad Prism 9. Comparisons between groups were performed using two-way ANOVA with Tukey’s or Dunnett’s *post hoc* test. P < 0.05 was defined as statistically significant. Results are shown as mean ± SEM.

### Ethics

NK cells were isolated from peripheral blood mononuclear cells (PBMCs) from voluntary blood donors with informed consent at the Finnish Red Cross Blood Service (Helsinki, Finland) and handled under an approval by the Ethics Committee, Hospital District of Northern Savo (1480/13.02.00/2019). These cells were isolated from buffy coats not required for treatment of patients. All in all, the buffy coats of 25 donors were used during the studies. Donor information is shown in **Supplementary Tables 5**, **6**.

## Results

### Selection method and cytokine timing affects final purity and expansion of NK cells

We studied 39 culture conditions with the aim of identifying an optimal expansion method for pure and cytotoxic NK cells. These culturing conditions (**Supplementary Figure 1**) comprised of three selection methods [Depletion of CD3+ cells (CD3-), enrichment of CD56+ cells (CD56+), and their combination (CD3-/CD56+)]; four activation protocols [continuous (Cont), 16-18 h pre-activation (Pre), pre-activation followed by re-activation on day 15 (Re), and culturing in IL-15 followed by short activation on day 15 (Boost)], and four cytokine combinations (IL-2/15, IL-21/15, IL-27/18/15 and IL-12/18/15). At the end of the culture (day 16), the cells were analyzed for their viability, expansion rate, purity, phenotype, and cytotoxicity against the U87-wt glioma cell line.

**Figure 1:**
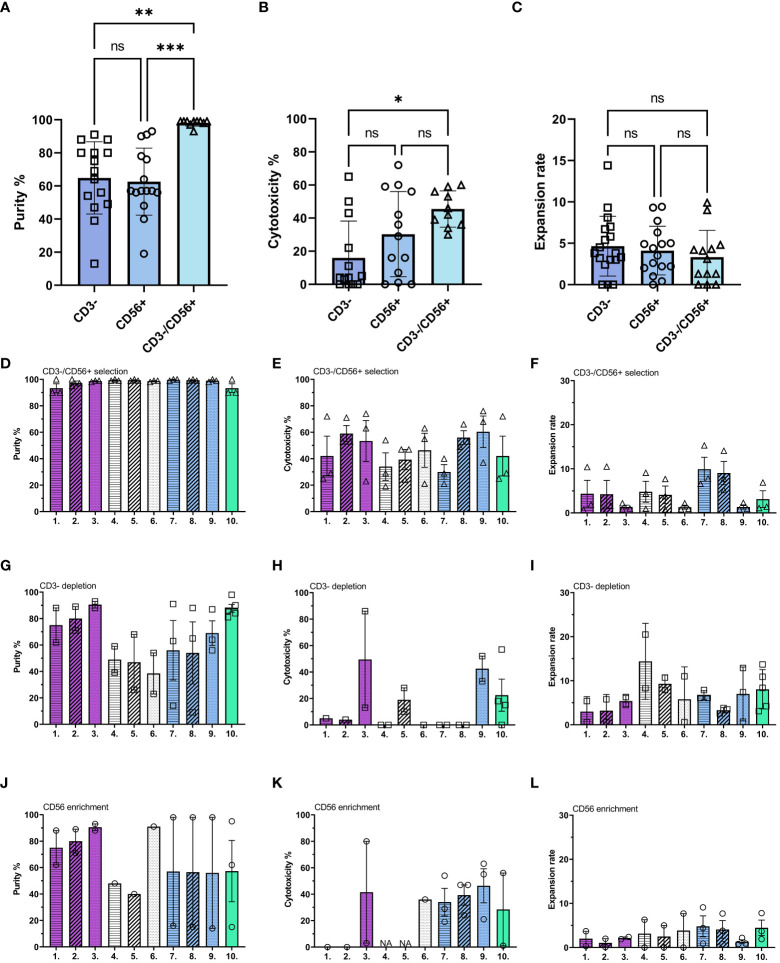
Comparison between different selection methods and cytokine combinations. NK cells were analyzed after 16 days expansion for their expansion rate, purity, and cytotoxicity against U87-wt glioblastoma cell line. **(A–C)** Comparison between three selection methods [depletion of CD3+ cells (CD3-, square), enrichment of CD56+ cells (CD56+, circle), and depletion of CD3+ cells followed by enrichment of CD56+ cells (CD3-/CD56+, triangle)]. **(A)** Purity of CD45+/CD3-/CD56+ NK cells in the population after ex vivo expansion. **(B)** Cytotoxicity against U87-wt with 1:1 E:T ratio. **(C)** Expansion rate calculated by dividing the output number of expanded NK cells after 16 days of culture by the NK cell number on day 0. **(D–L)** Comparison of different expansion methods (1-10; **Supplementary Table 3**) within the selection methods. Each symbol represents one expansion, all together 25 donors were used, minimum 2 donors per condition. If one datapoint or NA the measurement was not performed due to low yield in that expansion. Numbers 1-10 represent the different expansion conditions (1.IL-21/15 Pre, 2.IL-21/15 Re, 3.IL-21/15 Boost, 4.IL-27/18/15 Pre, 5.IL-27/18/15 Re, 6.IL-27/18/15 Boost, 7.IL-12/18/15 Pre, 8.IL-12/18/15 Re, 9.IL-12/18/15 Boost, 10.IL-2/15 Cont; Pre=pre-activation, Re, re-activation; Boost, activation on day 15; Cont, Continuous). Pattern indicates the activation timing: Pre-activation = horizontal stripes, Re-activation = diagonal stripes, Boost = dotted, Continuous = blank. Color indicates the cytokine combination: IL-21/15 = purple, IL-27/18/15 = white, IL-12/18/15 = blue, IL-2/15 = green. NA, Not available. Data shown as mean ± SEM. ***p< 0.001, **p< 0.01, *p< 0.05, ns, non significant.

First, we studied the impact from the selection methods to the expansion, cytotoxicity, and end purity. These results were used to determine the optimal starting material for the subsequent studies. After selection, the cells were expanded with different culture conditions (**Supplementary Figure 1**). CD3-/CD56+ selection resulted in the highest end purity and cytotoxicity with all activation protocols (**Figures 1A, B**; **Supplementary Table 5**), no significant differences between expansion ratios were detected between the selection methods (**Figure 1C**). Next, we compared purities (**Figures 1D, G, J**) cytotoxicity (**Figures 1E, H, K**) and expansion rate (**Figures 1F, I, L**) within the selection method. CD3-/CD56+ selection method produces most pure NK cells (**Figure 1D**) while CD3 depletion (**Figure 1G**) and CD56 enrichment (**Figure 1J**) had more variation between expansion methods. Highest overall cytotoxicities were gained with CD3-/CD56+ selection (**Figure 1E**) compared to CD3 depletion (**Figure 1H**) and CD56 enrichment (**Figure 1K**). Expansion rates were overall highest with the CD3 depletion (**Figure 1I**) and lowest with CD56 enrichment (**Figure 1L**). No significant differences were observed within the selection methods due to donor variation. Cytotoxicity was increased with pre-activation and boost with all conditions and was most prominent with CD3-/CD56+ selection (**Figure 1E**). Unlike the other cytokine combinations tested, which resulted in poor survival and expansion if continuously present, the IL-2/15 combination was tolerated continuously during the expansion (**Figures 1D–L**, condition 10). Other continuous protocols were omitted due to low cell viability and expansion rates. At this stage, one selection method (CD3-/CD56+) and 10 culturing conditions (**Supplementary Figure 2**, **Supplementary Table 3**) from the initial conditions, were selected for subsequent experiments based on the purity and expansion rate.

### NK cell phenotype is altered by the activation method

**Figure 2:**
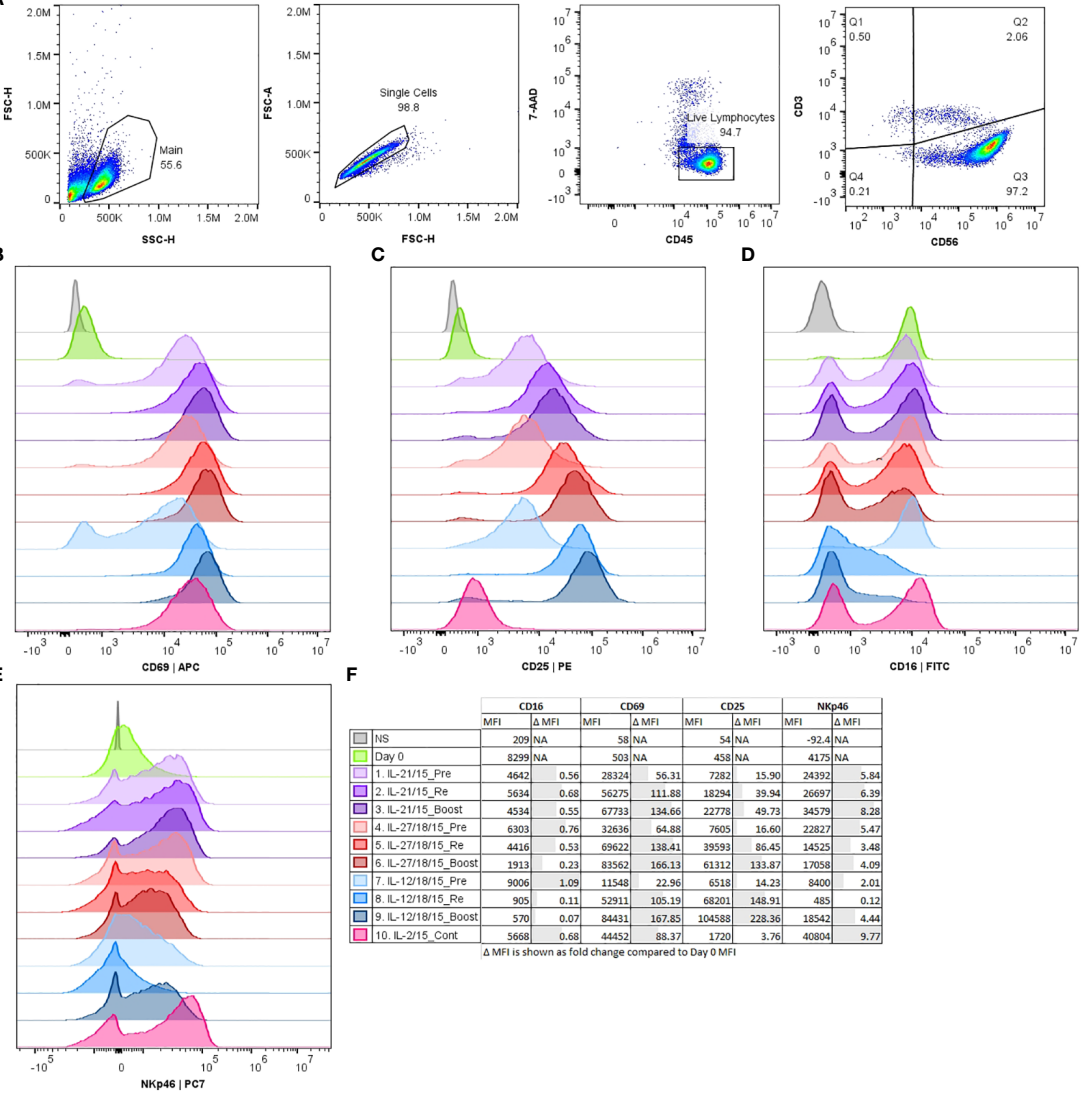
Histograms of flow cytometry data comparing expression of phenotype markers CD69, CD25, CD16, and NKp46 after CD3-/CD56+ selection (Day 0) and 16 days expansion with different expansion protocols (1-10). **(A)** Representative example from the gating strategy for viable (7-AAD-) CD45+/CD3-/CD56+ NK cells. Representative histograms shown from a single donor **(B–E)**. **(B)** CD69 expression, **(C)** CD25 expression, **(D)** CD16 expression, **(E)** NKp46 expression. **(F)** Group details and median fluorescence intensity (MFI) of the measured markers. Pre, pre-activation; Re, re-activation; Boost, activation on day 15; Cont, Continuous; NS, Non stained.

NK cells were identified as CD3 negative cells and CD56 positive (CD3-CD56+) within the CD45+ leukocyte population (**Figure 2A**). We noticed that the CD56 expression shifts from dim to bright during the expansion (**Supplementary Figure 3**). This was seen with all methods. To examine further how the used cytokine and activation methods alter the expression of known NK cell markers, we characterized the expression of CD69, CD25, CD16, and NKp46. The markers CD69 and CD25 represent NK cell activation and maturation, CD16 represents the ADCC activity and NKp46 the natural cytotoxicity (**Figures 2B–F**; **Supplementary Figure 4**).

From the measured NK markers the CD69 expression was induced during the expansion in all conditions being highest after boosting with all cytokine combinations (IL-2/15, IL-21/15, IL-27/18/15, IL-12/18/15) and lowest with IL-12/18/15 pre-activation (**Figure 2B**; **Supplementary Figure 4**). CD25 expression was upregulated with all methods except continuous IL-2/15 being lowest after pre-activation and highest after boosting (**Figure 2C**). IL-12/18/15 cytokine combination had the most profound impact on the phenotype by decreasing CD16 and NKp46 expression. The CD16 downregulation was most evident with IL-12/18/15 re-activation and boost (**Figure 2D**, conditions 8 and 9). CD16 downregulation was also seen to some extent when NK cells were boosted with IL-27/18/15 (**Figure 2D**, condition 6) compared to pre-activation with the same cytokines. NKp46 expression was lowest with IL-12/18/15 re-activation (**Figure 2E**, condition 8) when compared to other combinations (conditions 1-7, 9-10).

### Cytokines and expansion rate alter the NK cell cytotoxicity

**Figure 3:**
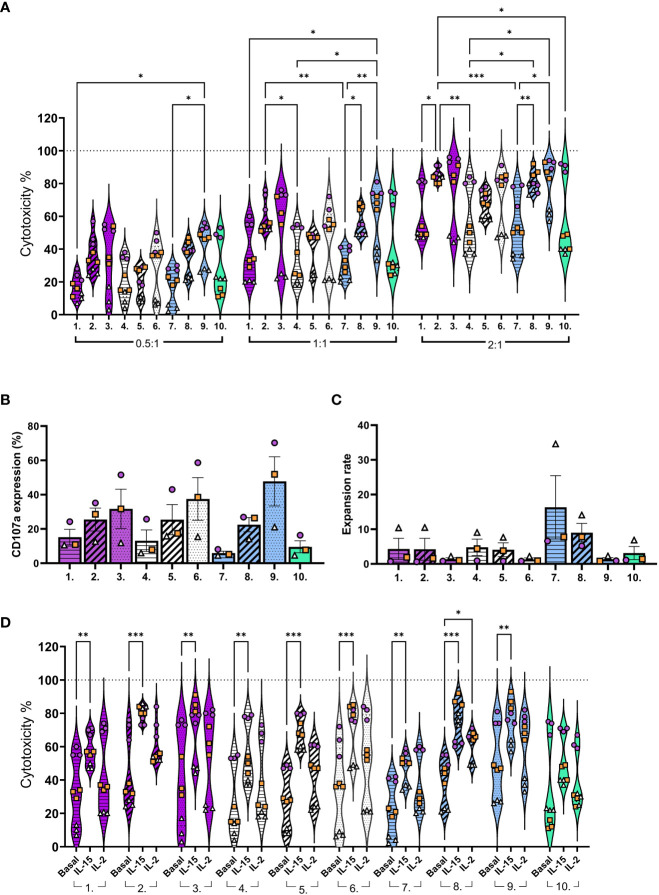
NK cell functionality against the glioblastoma cell line U87-wt after expansion with different conditions (1-10). **(A)** NK cell cytotoxicity with 0.5:1, 1:1, or 2:1 E:T ratios. **(B)** CD107a expression (degranulation) on CD45+ cells after 3 hour co-culturing (1:1 E:T). **(C)** Expansion rate calculated by dividing the output number of expanded NK cells after 16 days of culture by the NK cell number on day 0. **(D)** NK cell cytotoxicity (basal) compared to cytotoxicity with additional IL-2 (20 IU/ml) or IL-15 (5 ng/ml) in the co-cultures (1:1 E:T). E:T, Effector to target ratio. Numbers 1-10 represent the different expansion conditions (1.IL-21/15 Pre, 2.IL-21/15 Re, 3.IL-21/15 Boost, 4.IL-27/18/15 Pre, 5.IL-27/18/15 Re, 6.IL-27/18/15 Boost, 7.IL-12/18/15 Pre, 8.IL-12/18/15 Re, 9.IL-12/18/15 Boost, 10.IL-2/15 Cont; Pre=pre-activation, Re, re-activation; Boost, activation on day 15; Cont, Continuous). Pattern indicates the activation timing: Pre-activation = horizontal stripes, Re-activation = diagonal stripes, Boost = dotted, Continuous = blank. Color indicates the cytokine combination: IL-21/15 = purple, IL-27/18/15 =white, IL-12/18/15 = blue, IL-2/15 =green. Data are shown from 3 donors with three technical replicates, identified by symbol shape and color. ***p< 0.001, **p< 0.01, *p< 0.05.

For the next set of studies NK cells, after CD3-/CD56+ selection, were expanded with 10 different expansion methods (conditions (1-10); **Supplementary Figure 2**; **Supplementary Table 3**). The NK cell cytotoxicity was assessed against glioblastoma cell line U87wt. Donor variation in cytotoxicity was observed with most of the activation methods. This variation was lowest with IL-21/15 and IL-12/18/15 re-activations with higher effector to target (E:T) ratios (2:1) (**Figure 3A**, conditions 2 and 8; **Supplementary Table 4**). The pre-activated (Pre, conditions 1, 4 and 7) NK cells showed lower while the boosted (Boost; conditions 3, 6 and 9) exhibited higher cytotoxicity in response to all cytokine combinations (**Figure 3A**; **Supplementary Table 4**). This finding correlated with the NK cell degranulation, as measured by lysosome associated membrane protein-1 (CD107a) expression, after expansion and co-culturing with tumor targets (**Figure 3B**). Our results show a trend that high expansion rates can decrease the NK cell cytotoxicity. This was seen particularly with IL-12/18/15 pre-activation (**Figure 3C**, conditions 7). This phenomenon could be obscured in other conditions due to the low expansion rates or be specifically related to the IL-12/18/15 combination, or due to donor variation (**Figure 3C**).

As the expansion rate and method impacted the cytotoxicity we wanted to see if NK cells would respond to additional cytokines after *ex vivo* expansion. To study this, we added IL-2 or IL-15 with the NK cells to the co-cultures. Additional IL-15 (5 ng/ml) enhanced the cytotoxicity of all, except continuous IL-2/15 (**Figure 3D**, condition 10) expanded NK cells, whereas IL-2 (20 IU/ml) increased the cytotoxicity only for the IL-12/18/15 re-activated NK cells (**Figure 3D**, condition 8). As seen in the phenotype, the continuous IL-2/15 generated NK cells with low CD25 expression (**Figure 2C**, condition 10), which could explain the differences in NK cell responsiveness to additional IL-2.

**Figure 4:**
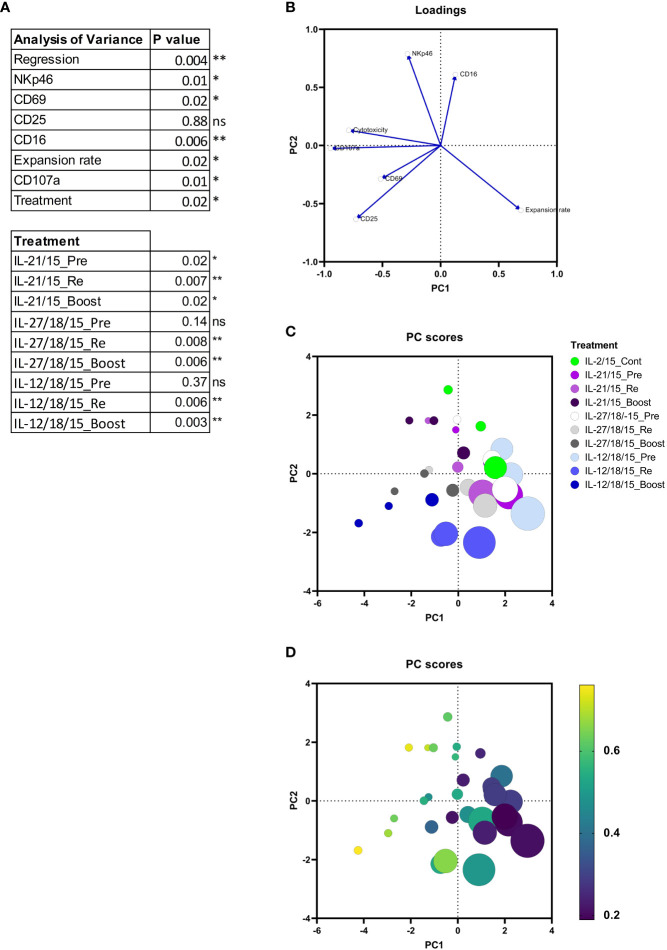
Multiple linear regression and Principal components analysis (PCA) from NK cell expansion rates, purity, cytotoxicity and phenotype markers (CD16, CD25, CD69, and NKp46), and degranulation (CD107a). **(A)** Multiple linear regression. The cytotoxicity of continuous IL-2/15 expanded NK cells was considered as a reference level. **(B)** Eigenvectors showing the variance in the input data. **(C)** Principal component (PC) scores indicating the expansion protocol and cytokines by color and the expansion rate by size. **(D)** PC indicating the cytotoxicity in color scale, yellow being the highest cytotoxicity rate and the expansion rate by size. Data collected from 3 donors. **p< 0.01, *p< 0.05, ns= nonsignificant.

To determine how the measured markers contribute to the cytotoxicity, we conducted a multiple regression analysis and a principal component analysis (PCA) from the following parameters: activation protocol, expansion rate, phenotype markers (CD25, CD69, CD16, NKp46), and CD107a (**Figure 4A**). Continuous IL-2/15 protocol was used as a reference level for the multiple linear regression analysis. It can be concluded that by multiple regression, using least-squares, the expression of CD107a, CD69, NKp46, and CD16, the activation protocol (treatment), and the expansion rate are significant measures of the variability in cytotoxicity. Furthermore, the pre-activation with IL-21/15 (IL-21/15_Pre), re-activation with IL-21/15, IL-27/18/15, or IL-12/18/15 (IL-21/15_Re, IL-27/18/15_Re, IL-12/18/15_Re), and boosting with IL-27/18/15 or IL-12/18/15 (IL-27/18/15_Boost, IL-12/18/15_Boost) increase the cytotoxicity significantly when compared to continuous IL-2/15 (IL-2/15_Cont) (**Figure 4A**). The PCA plots (**Figures 4B–D**) reveal the correlations between these parameters. They show that cytotoxicity correlates positively with CD107a and the phenotype markers CD25, CD69, CD16, and NKp46, and negatively with the expansion rate (eigenvectors in **Figure 4B**). The highest expansion rates were achieved with the IL-12/18/15 pre-activation (**Figure 4C**, light blue) while re-activation (Re, blue) increased the cytotoxicity (**Figure 4D**). Highest cytotoxicity, as already shown in **Figure 3**, was achieved with IL-21/15 and IL-12/18/15 boosting (**Figure 4D**, color scale shows the cytotoxicity), however, with modest expansion rates (**Figure 4C**, size indicates the expansion rate).

### Activation timing during expansion impacts the NK cell cytotoxicity

**Figure 5:**
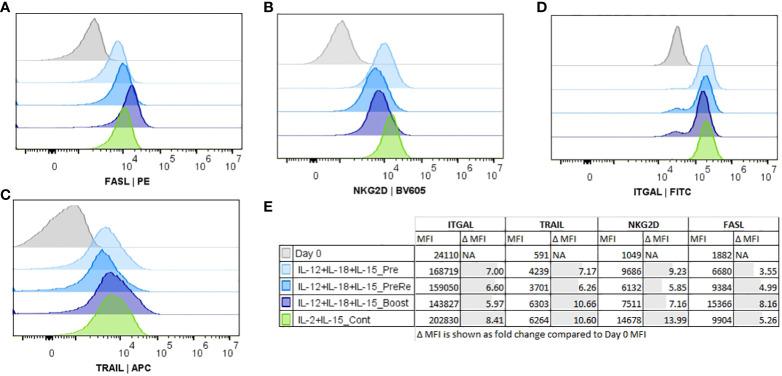
Comparison of histograms for the functionality markers Fas-ligand (FASL), natural killer group 2D (NKG2D), TNF-related apoptosis-inducing ligand (TRAIL), and lymphocyte function-associated antigen 1 (ITGAL) expression on CD45+/CD3-/CD56+ NK cells. NK cells, CD56+/CD3- selected, were expanded for 16 days with IL-12/18/15 (pre, re, and boost) and IL-2/15 (Cont). Representative histograms shown from a single donor **(A–D)**. **(A)** FASL-expression. **(B)** NKG2D expression. **(C)** TRAIL expression. **(D)** ITGAL expression. **(E)** Group details and median fluorescence intensity (MFI) of the measured markers. Pre, Pre-activation; Re, Re-activation; Boost, cytokine activation at the end of expansion. Cont, Continuously.

**Figure 6:**
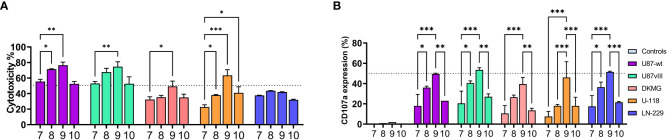
NK cell functionality against five glioma cell lines: U87-wt, U87vIII, DK-MG, U-118, and LN-229 after 16 days expansion with IL-12/18/15 (pre, re, and boost) and IL-2/15 (Cont). **(A)** NK cell cytotoxicity at 1:1 E:T ratio against the glioma cell lines. **(B)** CD107a expression (degranulation) on CD45+ cells after 3 hour co-culturing (1:1 E:T) with the glioma cell lines. Controls represent the basal expression level without co-culturing. Numbers 7-10 represent the different expansion conditions (7.IL-12/18/15 Pre, 8.IL-12/18/15 Re, 9.IL-12/18/15 Boost, 10.IL-2/15 Cont; Cont; Pre, pre-activation; Re, re-activation; Boost, activation on day 15; Cont, Continuous). E:T, Effector to target ratio. Data from two donors with three technical replicates represented as the mean ±SEM. ***p< 0.001, **p< 0.01, *p< 0.05.

At the final part of our study, we focused on the activation timing (Pre, Re, Boost). These studies were performed with the combination of IL-12/18/15, which had highest expansion rate within the cytokine combinations (**Figure 3C**, conditions 7-8), and continuous IL-2/15 was used as a control condition. We also measured the expression of additional NK markers (**Figures 5A–E**; **Supplementary Figure 5**) FasL, NKG2D, TRAIL, and ITGAL. Boosting induced the highest expression of TRAIL (**Figure 5C**) and FasL (**Figure 5A**), while IL-12/18/15 pre-activation and continuous IL-2/15 induced the highest expressions of NKG2D (**Figure 2B**) and ITGAL (**Figure 5D**). NK cell functionality was measured with cytotoxicity (**Figure 6A**) and degranulation (CD107a expression, **Figure 6B**) against five glioma cell lines (U87-wt, U87vIII, DK-MG, LN229 and U-118). Also, the cytokine secretion profile was assessed against two cell lines (U-87-wt and U-118). Degranulation increased in response to re-activation and boosting, being highest after boosting (**Figures 6A, B**, conditions 8 and 9). This finding was verified with degranulation related cytokine secretion profile (**Supplementary Figure 6**; IFN-g, GM-CSF) against U87-wt and U-118 cell lines. Cytotoxicity increased after re-activation and boost, as did the degranulation, against all cell lines, except LN-229 (**Figure 6A**, conditions 8-9). The cytotoxicity of IL-12/18/15 (re-activated; condition 8) and IL-2/15 (continuous; condition 10) expanded NK cells varied between the cell lines. U87-wt and U87vIII were more sensitive to IL-12/18/15 re-activated NK cells, while DK-MG, and U-118 cell lines responded better to IL-2/15 expanded NK cells (**Figure 6A**, conditions 8 and 10).

Since the differences in the target cell killing were evident, we characterized the expression of certain cytotoxicity-associated ligands from the target cell lines in an attempt to explain the differences in their sensitivity. We measured expression of the NKG2D ligands (UL16 binding protein (ULPB1) and MHC class I chain-related glycoproteins A and B (MICA and B)), LFA-1 ligand (ICAM-1), TRAIL and TRAIL receptors 1 and 2 (TRAILR1/2) and Fas ligand (FasL), and expression of CD56, CD45, EGFR and EGFRvIII. All target cell lines were positive for Fas and TRAILR1/2 (**Supplementary Table 2**). The expression of other markers varied between cell lines but could not explain the differences in the sensitivity. For example, the U-118 cell line, which was one of the most resistant targets, had the lowest expression of MICA/B and ULPB1. Another resistant cell line, LN229, had a similar expression profile to the most sensitive cell line, U87-wt.

## Discussion

Advances in chemotherapy, radiation, and neurosurgical procedures have increased survival of patients with brain tumors, but curative treatments are not yet available to malignancies like glioblastoma due to their recurrence and/or progression. NK cell-based therapies have shown efficacy in pre-clinical studies making them a promising treatment against glioblastoma (31, 32). NK cell-based immunotherapies provide an allogeneic “off-the-shelf” alternative to T cell therapy since they do not cause graft-versus-host disease. To fulfill these promises in the field of cancer immunotherapy, optimized *ex vivo* expansion methods need to be developed. Currently, most NK expansion methods are based on using cytokines with or without feeder cells. However, the use of feeder cells raises regulatory concerns. Even with sufficient irradiation of the feeder cells, the final cell therapy product requires an elimination step for the possible hazardous cells in order to clear the product from the genetic material and other components originating from the feeder cell line (33). Cytokine-based strategies are considered safer, but they come with the downfall of low NK cell yields.

During these studies we explored different cytokine-based methods to produce pure, functional NK cells from human peripheral blood. Our findings suggest that the proliferation capacity and functionality of NK cells is influenced by multiple factors, including the donor, cytokine combinations during cell culture, the timing of activation, and the starting material. As the development, proliferation and functionality of NK cells are controlled by multiple cytokines and growth factors (12, 34), it is critical to understand their impact on the NK cell manufacturing process. Recent discoveries have shown that NK cells can differentiate into a memory phenotype, improving the long-term anti-tumor responses (35–37). These memory-like NK cells develop as a response to foreign antigens, such as those of the human cytomegalovirus (CMV), or to cytokines IL-12, IL-15, and IL-18. This set of cytokines is known to enhance NK cell functional responses and has been harnessed for cancer immunotherapies (38, 39). Also in the current study, this combination of cytokines produced the highest amounts of pure NK cells. Pre-activation was important for the purity and expansion rate, while re-activation increased the cytotoxicity and evened out the donor variation (**Figure 3A**). The most cytotoxic NK cells, with limited expansion rate, were produced with a short exposure to IL-12/18/15 or IL-21/15 at the end of the expansions, referred here as boosting. IL-21 boost has been introduced previously by Wagner et al. (27). They reported that IL-21 boost increases the cytotoxicity compared to continuous IL-15 or IL-21 exposure. In our studies, the NK cells did not survive in the continuous presence of IL-21/15, and we omitted this condition after pre-screening. IL-21 has been reported to trigger apoptosis and to diminish IL-15-based benefits, which might be the reason for the low yield with the continuous protocol in our studies (40, 41).

For quality control purposes, it is important to discern the functionality and phenotype of the final product. In an attempt to discover the changes in the phenotype induced by our expansion methods, we measured the expression of different activation markers and receptors. Activation receptors NKG2D and NKp46, and target cell death inducing death receptor ligands FasL and TRAIL were all upregulated after activation, as has been shown previously (27). Activation-related differences were observed in the levels of CD69, CD25 and CD16, and in the level of CD107a which indicates increased degranulation. All of these markers, except CD16, were increased with IL-12/18/15 pre- and re-activation and were highest with IL-12/18/15 boost. Our findings differ from Romee et al. (2012), where the authors state that IL-12/18/15 pre-activated human NK cells do not have enhanced degranulation after re-activation (25). According to our results, the degranulation, INF-g secretion, and cytotoxicity were increased by the re-activation with these cytokines. Re-activation and boosting increased cytotoxicity against all of the used target glioblastoma cell lines (U87-wt, U87vIII, DK-MG, and LN-229), although modest against LN-229. NKG2D is considered as one of the main NK activating receptors. In an attempt to understand our targets better we phenotyped the target cell lines for NKG2D ligands among other markers (CD56, EGFR, EGFRvIII, FAS, FasL, TRAIL, TRAIL-R1/R2, MICA/B, ULPB1, ICAM-1, CD45, and NKG2D; **Supplementary Table 2**). The target phenotyping showed variation in the expression levels of NKG2D ligands, but they did not correlate with the outcome. For example, LN-229, which was the least sensitive target, had a higher expression of ICAM-1 compared to U87-wt and higher levels of ICAM-1 compared to U-118 which were more vulnerable NK cells. We did not study all of the known NK activating receptors, or the role of inhibition receptors (such as KIR and NKG2A) in the current study. More studies on NK cell interactions with their targets, blocking of the receptor interactions, and more complex target phenotyping would be needed to thoroughly understand what makes some target cells more resistant to NK cells.

NK cells use the CD16 receptor to induce antibody-dependent cellular cytotoxicity (ADCC) in their targets (42). This receptor can be utilized to mediate the clinical activity of traditional monoclonal antibody and dual targeting antigen-based therapies (32). Our data show that the IL-12/18/15 re-activation and boost downregulated CD16 expression (**Figure 2D**, conditions 8-9), which was not seen with other cytokine combinations. Similar results have been published by Romee et al. (2013) who showed that CD16 expression is regulated by a disintegrin and metalloprotease-17 (ADAM17), and its shedding was linked to IL-12/18-mediated activation and stimulation from target cells (43). These results could indicate that IL-12/18/15 re-activated or boosted NK cells, with low CD16 expression, might not be efficient in ADCC and, therefore, not the ideal match for combinatory therapies with mAbs. In place, or parallel with monoclonal antibodies, the cytokines IL-15, and perhaps IL-2, could be administered to improve cytotoxicity. Co-administration of IL-2 with the NK cells has been shown to prolong the survival of mice with U87-wt-derived tumors (44). However, the use of IL-15 in the clinical settings is reported to be more efficient, since it is known also to promote the activation of CD4+ and CD8+ T cells without significant activation of regulatory T-cells (Treg) (45, 46). In the current study, we noticed that IL-15 was superior to IL-2 in enhancing the NK cell cytotoxicity *in vitro* with all used protocols, except the continuous IL-2/15 expanded NK cells. The low CD25 expression, as seen here with the IL-2/15 expanded NK cells, has an impact on the NK cells’ sensitivity to IL-2 (47, 48). This is possibly related to the continuous presence of soluble IL-2, which inhibits the expression of the high affinity IL-2 receptor and its alpha chain (CD25) (47, 48). Combination of IL-2 and IL-15 has traditionally been the most commonly used method of NK expansions (49). In our study, these cytokines expanded NK cells with a moderate purity, but their cytotoxicity was low and not improved by co-administered cytokines *in vitro*. If this were transferred to clinical settings it could be speculated to lead to decreased efficacy and lack of tumor control.

During this study, we identified a cytokine-based expansion method that produces pure and highly cytotoxic NK cells. The best results in terms of purity and expansion rate were achieved when the NK cells (CD3-/CD56+ population) were first selected from the starting material, herein peripheral blood mononuclear cells, and pre-activated with IL-12/18/15. This protocol is known to produce cytokine-induced memory-like NK cells (25). The functionality of these memory-like NK cells was enhanced by re-activation and with co-administered IL-2 or IL-15. For the future manufacturing of a cell therapy product, a donor selection would be advisable, since the cell expansion rate, even with this protocol, varied. Our results indicate a negative correlation between the expansion rate and cytotoxicity of NK cells. A similar correlation has been previously observed with Anti-NKp46 mediated NK cell expansions (50). This link between proliferation and cytotoxicity could be used for donor selection or for designing the NK cell therapeutic products. Those donors whose NK cells expand could be used to produce higher number of memory-like NK cells, with IL-12/18/15 pre-activation. Their cytotoxicity could be then enhanced with re-activation or by co-administrating additional cytokines during the infusion. Conversely, those donors whose NK cells are not robustly expanding could be used for a product that is only boosted with IL-12/18/15 or IL-21/15 before administration, in order to obtain high cytotoxicity. Our data shows that the amount of NK cells and their expansion capabilities does not need to be a downfall; it can be turned to our advantage by using the ideal cytokines and activation protocols.

Currently cytokine induced memory-like cells (IL-12/18/15 pre-activated) are being tested in clinical trials (51). The needed amount of NK cell infusion can range from 2×10^6^ to 5×10^7^ CD3−CD56+ cells per kilogram (16, 52), which would mean 1.5 x10^7^ to 37.5 x10^8^ cells per infusion for person weighting 75 kg. With the highest expansion rate gained during our studies (~45 fold) the amount of starting material (CD3−/CD56+) would range from 3.3x10^6^ to 83x10^6^ for one infusion, which would not be feasible in small scale for higher doses. To manufacture the needed cell numbers for allogeneic off-the-shelf NK product, a closed, and automated system with large-scale capabilities is needed. One large-scale approach is to expand the isolated NK cells in static culture before transferring them to rocking bioreactors, which can be used to expand higher cell densities (53). The flask scale expansion method cannot directly be compared to clinical scale manufacturing, but it can give guidelines for the used cytokines and expansion protocols. It is known that culture conditions have an effect to the cell’s phenotype, functionality, and yield (54, 55).

This current study provides insights on the impact of NK cell selection and cytokine activation for the development of NK cell manufacturing, both for research and clinical purposes. Improvements in understanding NK cell biology and their optimal manufacturing conditions can drive further development of NK cell-based therapies. We describe here the effect of selection methods, cytokines, and the timing of activation for a feeder cell-free production of functional NK cells. These findings can be used as preliminary process parameters for large scale process development towards clinical applications.

## Data availability statement

The raw data supporting the conclusions of this article will be made available by the authors, without undue reservation.

## Ethics statement

The studies involving humans were approved by Hospital District of Northern Savo (1480/13.02.00/2019). The studies were conducted in accordance with the local legislation and institutional requirements. The participants provided their written informed consent to participate in this study.

## Author contributions

MS, KS, MV, and TK contributed to conception and design of the study. MS, SK, and SW carried out the experiments and collected the data. MS, KS, AB, AK and SW analysis and interpretation of results. MS wrote the first draft of the manuscript. All authors contributed to the article and approved the submitted version.
