# Supplementary material for: Cytokines impact natural killer cell phenotype and functionality against glioblastoma in vitro
Source: Front Immunol. 2023 Sep 28;14:1227064. doi: 10.3389/fimmu.2023.1227064 (PMC10569479; doi:10.3389/fimmu.2023.1227064)
Supplement: Supplementary file 1 [file DataSheet_1.docx]

Supplementary Material

Cytokines impact natural killer cell phenotype and functionality against glioblastoma in vitro

**Minna Sivonen^1,2^, Katja Sirviö^1,2^, Satu Kaipainen^1^, Sara Wojchowski^1^, Anssi Kailaanmäki^1^, Aubrey Bailey^1^, Martin Villalba^*3,4^, Tuija Kekarainen^*1^**

*** Correspondence:** Tuija Kekarainen: [tuija.kekarainen@kct.fi](mailto:Tuija.kekarainen@kct.fi), Martin Villalba: [martin.villalba@inserm.fr](mailto:martin.villalba@inserm.fr)

# Supplementary methods

Cytokine secretion profile

Cytokine and growth factor secretion profiles were measured with MACSPlex Cytotoxic T/NK Cell Kit, human (cat. 130-125-800, Miltenyi Biotec) from cytotoxicity assay co-culture media (1:1 E:T ratio). The media was collected and frozen after co-culturing and thawed for the analysis. Assay was performed according to manufacturer’s protocol. Briefly, 12 different cytokines (GM-CSF, Granzyme B, IFN-γ, IL-2, IL-4, IL-6, IL-10, IL-17A, IL-21, MCP-1 (CCL2), Perforin, and TNF-α) were detected with specific capture beads and distinguished based on their different fluorescent intensity in flow cytometry. Flow cytometric analysis was performed with CytoFLEX S (Beckman Coulter) and data was analyzed with CytExpert and GraphPad Prism 9. Concentrations were interpolated in GraphPad Prism 9, using Asymmetric Sigmoidal, 5PL, curve fitting.

# Supplementary Figures and Tables

## Supplementary Figures


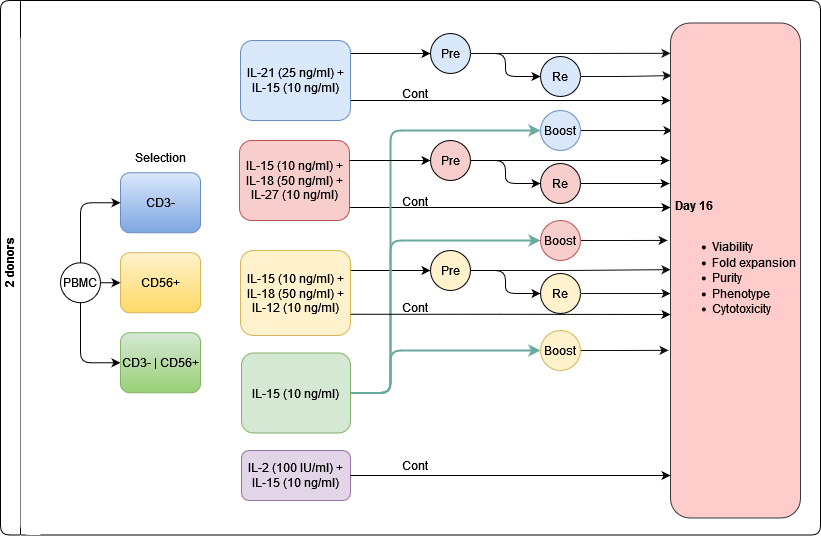


Supplementary Figure 1. The initial 39 activation protocols for cytokine-induced NK cell expansion. In the first part of the studies three selection methods (selection: CD3-, CD56+, CD3-/CD56+) were compared to select the most optimal method for the next part of the studies. These selection methods were then compared with five cytokine combinations: IL‑15, IL‑2/15, IL-21/15, IL‑27/18/15, and IL-12/18/15, and four activation methods (pre-activation (Pre), re-activation (Re), Boost (IL-15 continuous until boost) and continuous (Cont). After expansion all conditions were characterized by their cell viability, purity, expansion rate, phenotype, and functionality. PBMC = Peripheral blood mononuclear cells, CD3- = CD3 depletion, CD56+ = CD56 enrichment, CD3-/CD56+ = CD3 depletion followed by CD56 enrichment


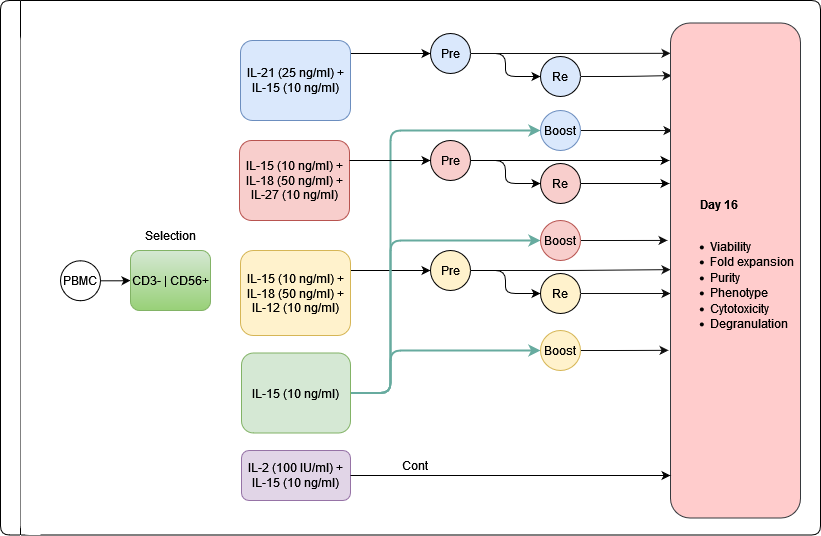


Supplementary Figure 2. Ten most optimal conditions were selected for the main studies. CD3‑/CD56+ selection with five cytokine combinations: IL-15, IL-12/18/15, IL-21/15, and IL‑27/18/15 with three activation methods (Pre-activation (Pre), Re-activation (Re), Boost (IL-15 continuous until boost) and IL-2/15 continuous (Cont). After expansion all conditions were characterized for cell viability, purity, expansion rate, phenotype, and functionality.


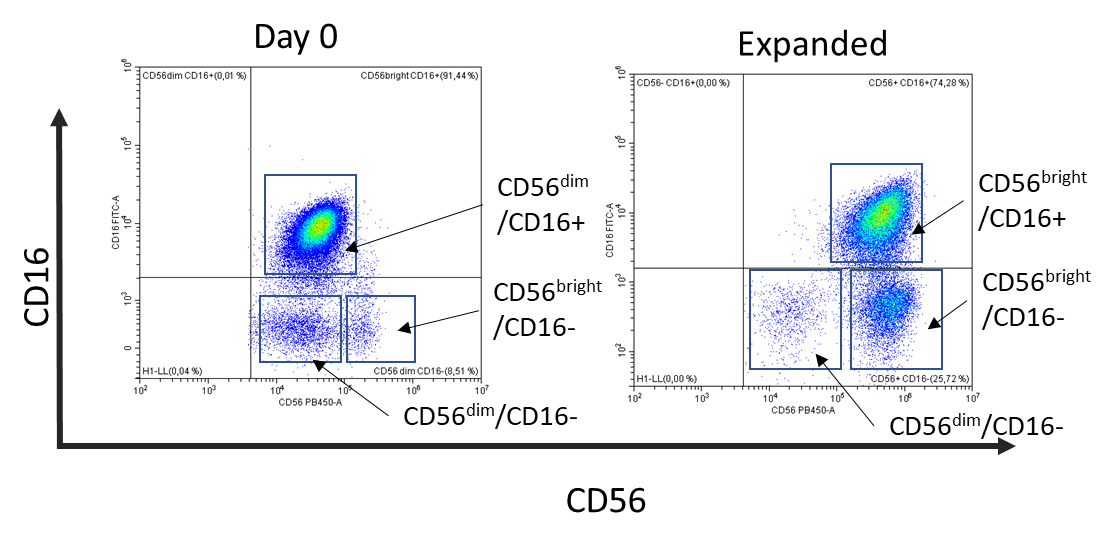


Supplementary Figure 3. Representative dot plots from changes in CD56 and CD16 expression during NK cell expansion. CD3-/CD56+ purified NK cells before (left) and after expansion with the IL-12/18/15 pre-activation protocol.


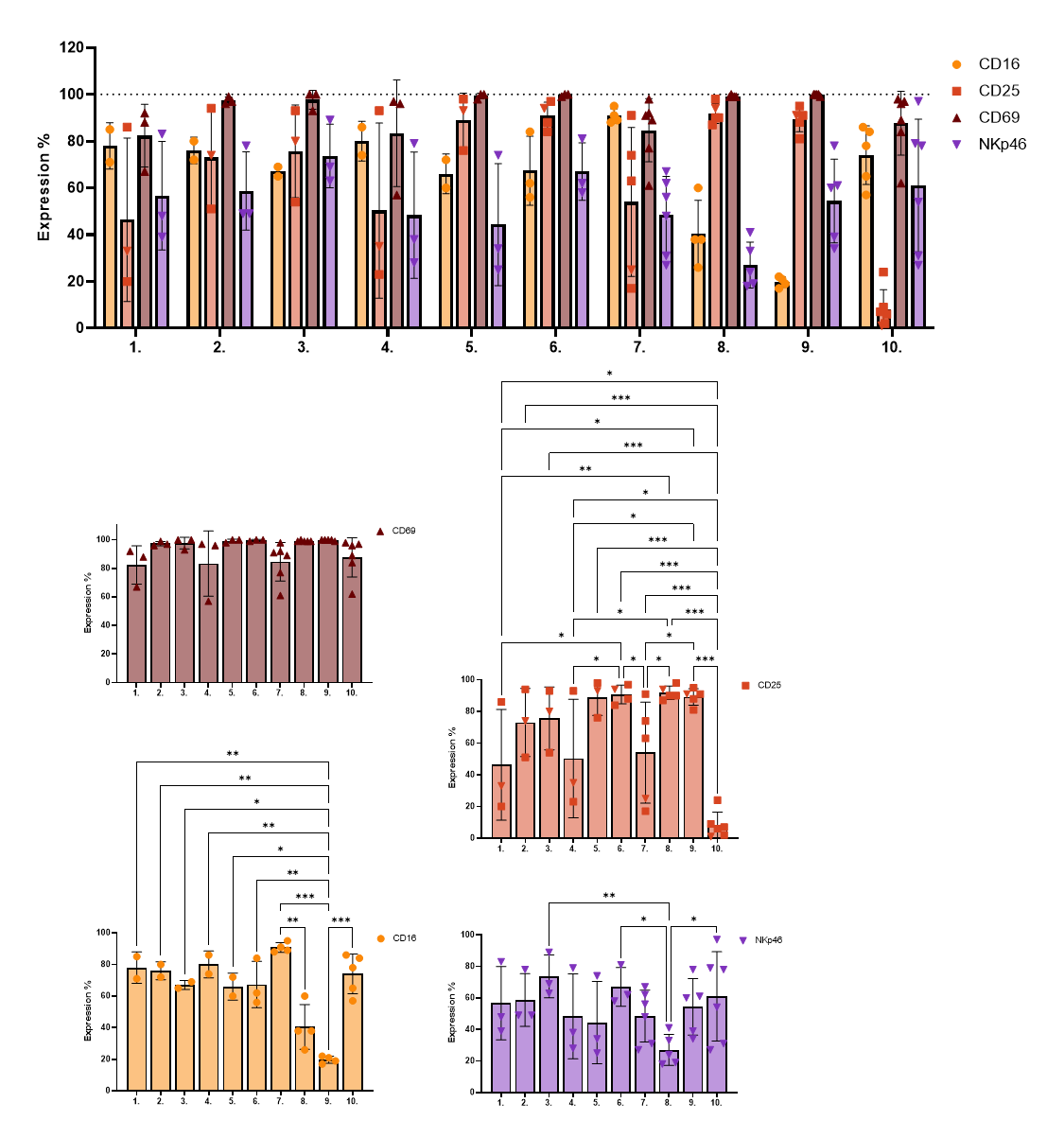


Supplementary Figure 4. CD69, CD25, CD16 and NKp46 expression on NK cells (CD45+/CD3-/CD56+). One symbol represents one donor. Numbers 1-10 represent the different expansion conditions (1.IL-21/15 Pre, 2.IL-21/15 Re, 3.IL-21/15 Boost, 4.IL-27/18/15 Pre, 5.IL-27/18/15 Re, 6.IL-27/18/15 Boost, 7.IL-12/18/15 Pre, 8.IL-12/18/15 Re, 9.IL-12/18/15 Boost, 10.IL-2/15 Cont; Cont; Pre=pre-activation, Re=re-activation, Boost=activation on day 15, Cont=Continuous; listed in Supplementary Table 3). Data shown as mean ±SEM. ***p < 0.001, **p < 0.01, *p < 0.05.

Supplementary Figure 5. FASL, NKG2D, TRAIL, and ITGAL expression on NK cells (CD45+/CD3-/CD56+). One symbol represents one donor. Numbers 7-10 represent the different expansion conditions (1.IL-21/15 Pre, 2.IL-21/15 Re, 3.IL-21/15 Boost, 4.IL-27/18/15 Pre, 5.IL-27/18/15 Re, 6.IL-27/18/15 Boost, 7.IL-12/18/15 Pre, 8.IL-12/18/15 Re, 9.IL‑12/18/15 Boost, 10.IL-2/15 Cont; Cont; Pre=pre-activation, Re=re-activation, Boost=activation on day 15, Cont=Continuous; listed in Supplementary Table 3). Data shown as mean ±SEM. ***p < 0.001, **p < 0.01, *p < 0.05.

Supplementary Figure 6. Cytokine secretion profiles against U-87 and U-118 glioblastoma cell lines. Co‑culture mediums from cytotoxicity assay were analyzed with MACSPlex Cytotoxic T/NK Cell Kit. Cytokine concentrations shown as pg/ml. Data from two donors, one shown with color and one parallel in gray. The red line shows the limit of detection. Numbers 7-10 represent the different expansion conditions (1.IL-21/15 Pre, 2.IL-21/15 Re, 3.IL-21/15 Boost, 4.IL-27/18/15 Pre, 5.IL‑27/18/15 Re, 6.IL-27/18/15 Boost, 7.IL-12/18/15 Pre, 8.IL-12/18/15 Re, 9.IL‑12/18/15 Boost, 10.IL-2/15 Cont; Cont; Pre=pre-activation, Re=re-activation, Boost=activation on day 15, Cont=Continuous; listed in Supplementary Table 3).

## Supplementary Tables

**Supplementary Table 1.** List of used antibodies for flow cytometry: specificity, clone name, conjugate and the provider.

| **Marker** | **Clone** | **Conjugate** | **Manufacturer** |
| --- | --- | --- | --- |
| **7-AAD** | NA | NA | BD Biosciences |
| **CD16** | REA423 | VB515 | Miltenyi Biotec |
| **CD25** | M-A251 | PE | BioLegend |
| **CD3** | OKT3 | BV510 | BioLegend |
| **CD45** | 2D1 | APC-Fire750 | BioLegend |
| **CD56** | 5.1H11 | BV421 | BioLegend |
| **CD69** | FN50 | APC | BioLegend |
| **CD107a** | REA792 | PE | Miltenyi Biotec |
| **EGFR** | S3 12D | APC | Absolute Antibody |
| **FASL** | NOK-1 | PE | BioLegend |
| **ICAM-1** | HA58 | APC | BioLegend |
| **ITGAL** | HI111 | FITC | BioLegend |
| **MIC-A/B** | 6D4 | PE | BioLegend |
| **NKG2D** | 1D11 | BV605 | BioLegend |
| **NKp46** | 900 | PECy7 | BioLegend |
| **TRAIL** | RIK-2 | APC | BioLegend |
| **ULPB-1** | 170818 | PE | BioLegend |

Supplementary Table 2. Comparison of expression of CD56, CD45, NKG2D, and Fas and Trail and their ligands, in different glioma cell lines (DK-MG, LN229, U-118, U-87-wt, U-87-vIII) and NK cells.

|  | **CD56** | **EGFR** | **EGFR vIII** | **FAS** | **FasL** | **TRAIL** | **TRAIL R1/2** | **MICA/B** | **ULBP1** | **ICAM-1** | **CD45** | **NKG2D** |
| --- | --- | --- | --- | --- | --- | --- | --- | --- | --- | --- | --- | --- |
| **DK-MG** | **-** | **+ (~30%)** | **+ (~30%)** | **+** | **-** | **-** | **+** | **+** | **+** | **+** | **+ (~50%)** | **-** |
| **LN229** | **+** | **-** | **-** | **+** | **-** | **-** | **+** | **+** | **+** | **+** | **-** | **-** |
| **U-118** | **+ (~70%)** | **+** | **-** | **+** | **-** | **-** | **+** | **+ (~20%)** | **+ (~5%)** | **+** | **-** | **-** |
| **U87- wt** | **+ (~40%)** | **+** | **-** | **+** | **-** | **-** | **+** | **+** | **+** | **+ (~50%)** | **-** | **-** |
| **U87vIII** | **NA** | **+** | **+** | **+** | **-** | **-** | **+** | **+** | **+** | **+** | **-** | **-** |
| **NK cell** | **+** | **-** | **-** | **+** | **-** | **+** | **-** | **-** | **-** | **+** | **+** | **+** |

**+** Indicates that all cells were positive to the specific marker; **(%)** indicates the part of the population positive for the marker; **-** Indicates that all cells were negative for the marker.

**Supplementary Table 3.** The 10 most optimal culturing conditions from CD3-/CD56+ cells, with the listed cytokine combinations.

|  | Activation | Cytokines |
| --- | --- | --- |
| 1 | Pre-activation (Pre) | IL-21/15 |
| 2 | Re-activation (Re) | IL-21/15 |
| 3 | Boost | IL-21/15 |
| 4 | Pre | IL-27/18/15 |
| 5 | Re | IL-27/18/15 |
| 6 | Boost | IL-27/18/15 |
| 7 | Pre | IL-12/18/15 |
| 8 | Re | IL-12/18/15 |
| 9 | Boost | IL-12/18/15 |
| 10 | Continuous (Cont) | IL-2/15 |

**Supplementary Table 4.** Statistics from Figure 4. Showing the values of mean, SEM and number of used donors (n).

|  | **1.** | | | **2.** | | | **3.** | | | **4.** | | | **5.** | | |
| --- | --- | --- | --- | --- | --- | --- | --- | --- | --- | --- | --- | --- | --- | --- | --- |
| **E:T** | Mean | SEM | n | Mean | SEM | n | Mean | SEM | n | Mean | SEM | n | Mean | SEM | n |
| 0.5:1 | 17 | 2 | 3 | 36 | 4 | 3 | 34 | 7 | 3 | 21 | 4 | 3 | 20 | 3 | 3 |
| 1:1 | 36 | 5 | 3 | 59 | 3 | 3 | 53 | 8 | 3 | 34 | 5 | 3 | 40 | 4 | 3 |
| 2:1 | 60 | 5 | 3 | 85 | 1 | 3 | 76 | 7 | 3 | 56 | 7 | 3 | 68 | 3 | 3 |
|  |  |  |  |  |  |  |  |  |  |  |  |  |  |  |  |
|  | **6.** | | | **7.** | | | **8.** | | | **9.** | | | **10.** | | |
| **E:T** | Mean | SEM | n | Mean | SEM | n | Mean | SEM | n | Mean | SEM | n | Mean | SEM | n |
| 0.5:1 | 29 | 6 | 3 | 18 | 4 | 3 | 35 | 3 | 3 | 43 | 4 | 3 | 28 | 6 | 3 |
| 1:1 | 47 | 7 | 3 | 30 | 3 | 3 | 56 | 3 | 3 | 60 | 6 | 3 | 43 | 7 | 3 |
| 2:1 | 72 | 6 | 3 | 54 | 6 | 3 | 81 | 2 | 3 | 80 | 5 | 3 | 58 | 8 | 3 |

**n=** number of used donors; Data is calculated from three technical replicates per donor; Numbers **1-10** represent the different expansion condition, described in Supplementary Table 3.

**Supplementary Table 5.**  The data showing the mean, SD and n from each preliminary screened condition.

|  |  | IL-2/15 | | | | | | | | |
| --- | --- | --- | --- | --- | --- | --- | --- | --- | --- | --- |
|  |  | Expansion | | | Purity | | | Cytotoxicity | | |
|  |  | Mean | SD | n | Mean | SD | n | Mean | SD | n |
| CD3- | Cont | 8.1 | 4.0 | 5 | 88% | 6% | 5 | 22% | 21% | 4 |
|  | Pre | 3.0 | 2.4 | 2 | 80% | 14% | 2 | 3% | NA | 1 |
|  | Re | 2.4 | 1.6 | 2 | 80% | 10% | 2 | NA | NA | NA |
|  | Boost | 3.8 | 2.0 | 2 | 88% | 2% | 2 | 65% | NA | 1 |
| CD56+ | Cont | 4.4 | 2.5 | 3 | 57% | 33% | 3 | 29% | 28% | 2 |
|  | Pre | 2.6 | 2.4 | 2 | 78% | 19% | 2 | 0% | NA | 1 |
|  | Re | 2.2 | 2.0 | 2 | 76% | 20% | 2 | NA | NA | NA |
|  | Boost | 3.5 | 0.9 | 2 | 93% | 3% | 2 | 72% | NA | 1 |
| CD3-/CD56+ | Cont | 3.1 | 2.7 | 3 | 93% | 4% | 3 | 42% | 21% | 3 |
|  | Pre | NA | NA | NA | NA | NA | NA | NA | NA | NA |
|  | Re | NA | NA | NA | NA | NA | NA | NA | NA | NA |
|  | Boost | NA | NA | NA | NA | NA | NA | NA | NA | NA |
|  |  |  |  |  |  |  |  |  |  |  |
|  |  | IL-21/15 | | | | | | | | |
|  |  | Expansion | | | Purity | | | Cytotoxicity | | |
|  |  | Mean | SD | n | Mean | SD | n | Mean | SD | n |
| CD3- | Cont | 3.8 | 1.2 | 2 | 64% | 6% | 2 | NA | NA | NA |
|  | Pre | 3.0 | 2.4 | 2 | 75% | 13% | 2 | 5% | NA | 1 |
|  | Re | 3.2 | 2.6 | 2 | 80% | 9% | 2 | 4% | NA | NA |
|  | Boost | 5.4 | 0.9 | 2 | 91% | 3% | 2 | 50% | 37% | 2 |
| CD56+ | Cont | 0.4 | 0.2 | 2 | 19% | 16% | 2 | 7% | NA | 1 |
|  | Pre | 2.0 | 1.7 | 2 | 64% | 16% | 2 | 0% | NA | 1 |
|  | Re | 1.0 | 0.9 | 2 | 57% | 19% | 2 | 1% | NA | 1 |
|  | Boost | 2.2 | 0.3 | 2 | 90% | 3% | 2 | 42% | 39% | 2 |
| CD3-/CD56+ | Cont | 0** | 0.0 | 2 | NA | NA | NA | NA | NA | NA |
|  | Pre | 4.3 | 4.3 | 3 | 98% | 1% | 3 | 36% | 15% | 3 |
|  | Re | 4.2 | 4.4 | 3 | 97% | 2% | 3 | 59% | 8% | 3 |
|  | Boost | 1.3 | 0.6 | 3 | 99% | 1% | 3 | 53% | 22% | 3 |
|  |  |  |  |  |  |  |  |  |  |  |
|  |  | IL-27/18/15 | | | | | | | | |
|  |  | Expansion | | | Purity | | | Cytotoxicity | | |
|  |  | Mean | SD | n | Mean | SD | n | Mean | SD | n |
| CD3- | Cont | 4.5 | 4.4 | 2 | 13% | 4% | 2 | 11% | NA | 1 |
|  | Pre | 14.4 | 6.1 | 2 | 49% | 10% | 2 | 0% | 0% | 2 |
|  | Re | 9.3 | 1.4 | 2 | 47% | 21% | 2 | 4% | NA | NA |
|  | Boost | 5.8 | 5.2 | 2 | 39% | 15% | 2 | 0% | NA | 1 |
| CD56+ | Cont | 4.9 | NA | 1*** | 56% | NA | 1*** | NA | NA | 1*** |
|  | Pre | 6.3 | NA | 1*** | 48% | NA | 1*** | NA | NA | 1*** |
|  | Re | 5.0 | NA | 1*** | 40% | NA | 1*** | NA | NA | 1*** |
|  | Boost | 7.7 | NA | 1*** | 91% | NA | 1*** | 36% | NA | 1*** |
| CD3-/CD56+ | Cont | 0** | 0.0 | 2 | NA | NA | NA | NA | NA | NA |
|  | Pre | 4.8 | 3.3 | 3 | 99% | 0% | 3 | 34% | 15% | 3 |
|  | Re | 4.1 | 2.8 | 3 | 99% | 0% | 3 | 39% | 11% | 3 |
|  | Boost | 1.3 | 0.6 | 3 | 99% | 1% | 3 | 46% | 18% | 3 |
|  |  |  |  |  |  |  |  |  |  |  |
|  |  | IL-12/18/15 | | | | | | | | |
|  |  | Expansion | | | Purity | | | Cytotoxicity | | |
|  |  | Mean | SD | n | Mean | SD | n | Mean | SD | n |
| CD3- | Cont | 0** | 0.0 | 2 | NA | NA | NA | NA | NA | NA |
|  | Pre | 6.7 | 0.9 | 3 | 56% | 32% | 0 | 0% | 0% | 2 |
|  | Re | 3.3 | 0.5 | 3 | 54% | 33% | 3 | 0% | 0% | 2 |
|  | Boost | 7.0 | 5.0 | 3 | 69% | 13% | 3 | 43% | 9% | 2 |
| CD56+ | Cont | 0** | 0.0 | 2 | NA | NA | NA | NA | NA | NA |
|  | Pre | 9.3 | 4.0 | 2 | 57% | 0.4 | 2 | 16% | NA | 1 |
|  | Re | 9.4 | 5.1 | 2 | 57% | 0.4 | 2 | NA | NA | NA |
|  | Boost | 4.9 | 2.0 | 2 | 56% | 0.4 | 2 | 16% | NA | 1 |
| CD3-/CD56+ | Cont | 0** | 0.0 | 2 | NA | NA | NA | NA | NA | NA |
|  | Pre | 9.9 | 3.9 | 3 | 99% | 0% | 3 | 30% | 8% | 3 |
|  | Re | 9.0 | 3.7 | 3 | 99% | 0% | 3 | 56% | 7% | 3 |
|  | Boost | 1.3 | 0.7 | 3 | 99% | 1% | 3 | 60% | 17% | 3 |

** No expansion; *** Only one donor expanded; NA=Not available, The cell amount after expansion was not enough to perform the cytotoxicity assay; n= number of used donors; Cytotoxicity against U87-wt with 1:1 E:T ratio; E:T =Effector to target ratio

Supplementary Table 6. Donor information per used selection and expansion method.

| IL-2/15 | **Don** | **Selec** | **Treat** | **Exp** | **Pur** | **Cyt** |  |  | **Don** | **Selec** | **Treat** | **Exp** | **Pur** | **Cyt** |
| --- | --- | --- | --- | --- | --- | --- | --- | --- | --- | --- | --- | --- | --- | --- |
|  | 1 | CD3- | Cont | 4.2 | 81% | 57% |  | IL-21/15 | 1 | CD3- | Cont | 2.6 | 70% | NA |
|  | 4 | CD3- | Cont | 10.9 | 90% | 18% |  |  | 4 | CD3- | Cont | 5.0 | 57% | NA |
|  | 10 | CD3- | Cont | 13.7 | 86% | 0% |  |  | 1 | CD3- | Pre | 0.6 | 62% | NA |
|  | 15 | CD3- | cont | 3.1 | 98% | 15% |  |  | 4 | CD3- | Pre | 5.4 | 88% | 5% |
|  | 16 | CD3- | cont | 8.4 | 84% | NA |  |  | 1 | CD3- | PreRe | 0.6 | 71% | NA |
|  | 1 | CD3- | Pre | 0.5 | 66% | NA |  |  | 4 | CD3- | PreRe | 5.8 | 89% | 4% |
|  | 4 | CD3- | Pre | 5.4 | 94% | 3% |  |  | 1 | CD3- | Boost | 6.3 | 88% | 86% |
|  | 1 | CD3- | PreRe | 0.8 | 70% | NA |  |  | 4 | CD3- | Boost | 4.5 | 93% | 13% |
|  | 4 | CD3- | PreRe | 4.0 | 90% | NA |  |  | 3 | CD56+ | Cont | 0.6 | 35% | 7% |
|  | 1 | CD3- | Boost | 5.9 | 90% | 65% |  |  | 6 | CD56+ | Cont | 0.2 | 3% | NA |
|  | 4 | CD3- | Boost | 1.8 | 86% | NA |  |  | 3 | CD56+ | Pre | 0.3 | 80% | NA |
|  | 2 | CD56+ | Cont | 1.9 | 62% | 56% |  |  | 6 | CD56+ | Pre | 3.7 | 49% | 0% |
|  | 5 | CD56+ | Cont | 7.8 | 95% | 1% |  |  | 3 | CD56+ | PreRe | 0.1 | 76% | NA |
|  | 11 | CD56+ | Cont | 3.7 | 15% | NA |  |  | 6 | CD56+ | PreRe | 2.0 | 37% | 1% |
|  | 2 | CD56+ | Pre | 0.2 | 59% | NA |  |  | 3 | CD56+ | Boost | 1.9 | 87% | 80% |
|  | 5 | CD56+ | Pre | 4.9 | 97% | 0% |  |  | 6 | CD56+ | Boost | 2.4 | 93% | 3% |
|  | 2 | CD56+ | PreRe | 0.2 | 57% | NA |  |  | 21 | CD3-\|CD56+ | Pre | 0.7 | 98% | 56% |
|  | 5 | CD56+ | PreRe | 4.2 | 96% | NA |  |  | 22 | CD3-\|CD56+ | Pre | 1.9 | 96% | 32% |
|  | 2 | CD56+ | Boost | 2.6 | 89% | 72% |  |  | 24 | CD3-\|CD56+ | Pre | 10.4 | 100% | 20% |
|  | 5 | CD56+ | Boost | 4.5 | 96% | NA |  |  | 21 | CD3-\|CD56+ | PreRe | 0.6 | 97% | 71% |
|  | 21 | CD3-\|CD56+ | cont | 1.1 | 90% | 72% |  |  | 22 | CD3-\|CD56+ | PreRe | 1.6 | 95% | 53% |
|  | 22 | CD3-\|CD56+ | cont | 1.4 | 90% | 25% |  |  | 24 | CD3-\|CD56+ | PreRe | 10.5 | 100% | 53% |
|  | 24 | CD3-\|CD56+ | cont | 6.9 | 100% | 29% |  |  | 21 | CD3-\|CD56+ | Boost | 0.8 | 99% | 74% |
|  |  |  |  |  |  |  |  |  | 22 | CD3-\|CD56+ | Boost | 1 | 98% | 63% |
|  |  |  |  |  |  |  |  |  | 24 | CD3-\|CD56+ | Boost | 2.2 | 99% | 23% |
|  |  |  |  |  |  |  |  |  |  |  |  |  |  |  |
|  | **Don** | **Selec** | **Treat** | **Exp** | **Pur** | **Cyt** |  |  | **Don** | **Selec** | **Treat** | **Exp** | **Pur** | **Cyt** |
| IL-27/18/15 | 7 | CD3- | Cont | 0.1 | 9% | NA |  | IL-12/18/15 | 7 | CD3- | Pre | 6.1 | 63% | 0% |
|  | 9 | CD3- | Cont | 9.0 | 17% | 11% |  |  | 10 | CD3- | Pre | 7.9 | 14% | NA |
|  | 7 | CD3- | Pre | 8.3 | 59% | 0% |  |  | 11 | CD3- | Pre | 6.1 | 91% | 0% |
|  | 9 | CD3- | Pre | 20.5 | 39% | 0% |  |  | 7 | CD3- | PreRe | 3.8 | 65% | 0% |
|  | 7 | CD3- | PreRe | 7.9 | 68% | 28% |  |  | 10 | CD3- | PreRe | 3.5 | 9% | NA |
|  | 9 | CD3- | PreRe | 10.7 | 26% | 10% |  |  | 11 | CD3- | PreRe | 2.6 | 88% | 0% |
|  | 7 | CD3- | Boost | 0.6 | 54% | NA |  |  | 7 | CD3- | Boost | 0.7 | 64% | NA |
|  | 9 | CD3- | Boost | 11.0 | 23% | 0% |  |  | 10 | CD3- | Boost | 12.9 | 56% | 52% |
|  | 8 | CD56+ | Cont | 4.9 | 56% | NA |  |  | 11 | CD3- | Boost | 7.4 | 87% | 33% |
|  | 8 | CD56+ | Pre | 6.3 | 48% | NA |  |  | 9 | CD56+ | Pre | 13.3 | 98% | 16% |
|  | 8 | CD56+ | PreRe | 5.0 | 40% | NA |  |  | 12 | CD56+ | Pre | 5.2 | 16% | NA |
|  | 8 | CD56+ | Boost | 7.7 | 91% | 36% |  |  | 12 | CD56+ | PreRe | 4.3 | 15% | NA |
|  | 21 | CD3-\|CD56+ | Pre | 0.9 | 99% | 54% |  |  | 9 | CD56+ | PreRe | 14.6 | 98% | 52% |
|  | 22 | CD3-\|CD56+ | Pre | 4.4 | 99% | 29% |  |  | 9 | CD56+ | Boost | 6.9 | 98% | 16% |
|  | 24 | CD3-\|CD56+ | Pre | 9.1 | 100% | 19% |  |  | 12 | CD56+ | Boost | 2.9 | 14% | NA |
|  | 21 | CD3-\|CD56+ | PreRe | 0.8 | 99% | 47% |  |  | 21 | CD3-\|CD56+ | Pre | 6.6 | 100% | 40% |
|  | 22 | CD3-\|CD56+ | PreRe | 3.8 | 99% | 47% |  |  | 22 | CD3-\|CD56+ | Pre | 7.8 | 99% | 29% |
|  | 24 | CD3-\|CD56+ | PreRe | 7.7 | 100% | 24% |  |  | 25 | CD3-\|CD56+ | Pre | 15.3 | 100% | 21% |
|  | 21 | CD3-\|CD56+ | Boost | 0.8 | 99% | 63% |  |  | 21 | CD3-\|CD56+ | PreRe | 5.2 | 99% | 53% |
|  | 22 | CD3-\|CD56+ | Boost | 0.9 | 98% | 55% |  |  | 22 | CD3-\|CD56+ | PreRe | 7.8 | 99% | 66% |
|  | 24 | CD3-\|CD56+ | Boost | 2.2 | 99% | 21% |  |  | 25 | CD3-\|CD56+ | PreRe | 14.1 | 100% | 49% |
|  |  |  |  |  |  |  |  |  | 21 | CD3-\|CD56+ | Boost | 0.9 | 99% | 76% |
|  |  |  |  |  |  |  |  |  | 22 | CD3-\|CD56+ | Boost | 0.8 | 98% | 68% |
|  |  |  |  |  |  |  |  |  | 25 | CD3-\|CD56+ | Boost | 2.3 | 100% | 37% |
|  | | | | | | | | | | |  |  |  |  |

NA =The cell amount after expansion was not enough to perform the cytotoxicity assay, Don = Donor number, Selec = used selection method, Treat = used expansion method, Exp = Expansion rate, Pur = Amount (%) of CD3-/CD56+ cells after expansion, Cyt. = Cytotoxicity against U-87-wt glioblastoma cell line.
